# Tenascin-C Potentiates Wnt Signaling in Thyroid Cancer

**DOI:** 10.1210/endocr/bqaf030

**Published:** 2025-02-14

**Authors:** Heather A Hartmann, Matthew A Loberg, George J Xu, Anna C Schwarzkopf, Sheau-Chiann Chen, Courtney J Phifer, Kailey Caroland, Hua-Chang Chen, Diana Diaz, Megan L Tigue, Amanda B Hesterberg, Jean-Nicolas Gallant, Sophia M Shaddy, Quanhu Sheng, James L Netterville, Sarah L Rohde, Carmen C Solórzano, Lindsay A Bischoff, Naira Baregamian, Paula J Hurley, Barbara A Murphy, Jennifer H Choe, Eric C Huang, Fei Ye, Ethan Lee, Vivian L Weiss

**Affiliations:** Department of Pathology, Microbiology, and Immunology, Vanderbilt University Medical Center, Nashville, TN 37232, USA; Department of Pathology, Microbiology, and Immunology, Vanderbilt University Medical Center, Nashville, TN 37232, USA; Department of Pathology, Microbiology, and Immunology, Vanderbilt University Medical Center, Nashville, TN 37232, USA; Department of Cell and Developmental Biology, Vanderbilt University, Nashville, TN 37232, USA; Department of Biostatistics, Vanderbilt University Medical Center, Nashville, TN 37232, USA; Department of Pathology, Microbiology, and Immunology, Vanderbilt University Medical Center, Nashville, TN 37232, USA; Department of Pathology, Microbiology, and Immunology, Vanderbilt University Medical Center, Nashville, TN 37232, USA; Department of Biostatistics, Vanderbilt University Medical Center, Nashville, TN 37232, USA; Department of Pathology, Microbiology, and Immunology, Vanderbilt University Medical Center, Nashville, TN 37232, USA; Department of Pathology, Microbiology, and Immunology, Vanderbilt University Medical Center, Nashville, TN 37232, USA; Department of Medicine, Vanderbilt University Medical Center, Nashville, TN 37232, USA; Department of Otolaryngology—Head & Neck Surgery, Vanderbilt University Medical Center, Nashville, TN 37232, USA; Department of Laboratory Medicine and Pathology, University of Washington School of Medicine, Seattle, WA 98195, USA; Department of Biostatistics, Vanderbilt University Medical Center, Nashville, TN 37232, USA; Department of Otolaryngology—Head & Neck Surgery, Vanderbilt University Medical Center, Nashville, TN 37232, USA; Department of Otolaryngology—Head & Neck Surgery, Vanderbilt University Medical Center, Nashville, TN 37232, USA; Department of Surgery, Vanderbilt University Medical Center, Nashville, TN 37232, USA; Department of Medicine, Vanderbilt University Medical Center, Nashville, TN 37232, USA; Department of Surgery, Vanderbilt University Medical Center, Nashville, TN 37232, USA; Department of Medicine, Vanderbilt University Medical Center, Nashville, TN 37232, USA; Department of Urology, Vanderbilt University, Nashville, TN 37232, USA; Department of Medicine, Vanderbilt University Medical Center, Nashville, TN 37232, USA; Department of Medicine, Vanderbilt University Medical Center, Nashville, TN 37232, USA; Department of Laboratory Medicine and Pathology, University of Washington School of Medicine, Seattle, WA 98195, USA; Department of Biostatistics, Vanderbilt University Medical Center, Nashville, TN 37232, USA; Department of Cell and Developmental Biology, Vanderbilt University, Nashville, TN 37232, USA; Department of Pharmacology, Vanderbilt University, Nashville, TN 37232, USA; Department of Pathology, Microbiology, and Immunology, Vanderbilt University Medical Center, Nashville, TN 37232, USA; Department of Cell and Developmental Biology, Vanderbilt University, Nashville, TN 37232, USA; Department of Otolaryngology—Head & Neck Surgery, Vanderbilt University Medical Center, Nashville, TN 37232, USA; Department of Pharmacology, Vanderbilt University, Nashville, TN 37232, USA

**Keywords:** thyroid cancer, tenascin-c, Wnt-2, invasion, metastasis

## Abstract

Tenascin-C (TNC) is a secreted extracellular matrix protein that is highly expressed during embryonic development and re-expressed during wound healing, inflammation, and neoplasia. Studies in developmental models suggest that TNC may regulate the Wnt signaling pathway. Our laboratory has shown high levels of Wnt signaling and TNC expression in anaplastic thyroid cancer (ATC), a highly lethal cancer with an abysmal approximately 3- to 5-month median survival. Here, we investigated the role of TNC in facilitating ligand-dependent Wnt signaling in thyroid cancer. We used bulk RNA-sequencing from 3 independent multi-institutional thyroid cancer patient cohorts. TNC expression was spatially localized in patient tumors with RNA in situ hybridization. The role of TNC was investigated in vitro using Wnt reporter assays and in vivo with a NOD.PrkdcscidIl2rg^–/−^ mouse ATC xenograft tumor model. TNC expression was associated with aggressive thyroid cancer behavior, including anaplastic histology, extrathyroidal extension, and metastasis. Spatial localization of TNC in patient tissue demonstrated a dramatic increase in expression within cancer cells along the invasive edge, adjacent to Wnt ligand–producing fibroblasts. TNC expression was also increased in areas of intravascular invasion. In vitro, TNC bound Wnt ligands and potentiated Wnt signaling. Finally, in an ATC mouse model, TNC increased Wnt signaling, tumor burden, invasion, and metastasis. Altogether, TNC potentiated ligand-driven Wnt signaling and promotes cancer cell invasion and metastasis in a mouse model of thyroid cancer. Understanding the role of TNC and its interaction with Wnt ligands could lead to the development of novel biomarkers and targeted therapeutics for thyroid cancer.

Thyroid cancer is projected to be the fourth leading cancer diagnosed in the United States by 2030 (1). Most thyroid cancers are well-differentiated tumors that are treatable with surgery and radioactive iodine. However, up to 30% of patients will have metastasis, recurrence, or progression (2, 3). Anaplastic thyroid carcinoma (ATC) is a highly lethal, aggressive form of thyroid cancer that grows quickly in the neck, leading to rapid airway compression. There has been minimal progress in treating patients with ATC, with a 12-month overall survival of less than 20% and metastasis being found at diagnosis in 50% of ATC patients (4). The high mortality rate of ATC is related to the rapid invasion of tumor cells into adjacent neck structures and distant metastasis of cancer cells into the lungs (5). An improved understanding of the mechanism of tumor cell invasion and metastasis could lead to targeted therapies and improve survival in ATC.

Technological advances, including DNA and RNA sequencing, have enhanced our understanding of many tumors, including those in the thyroid. Genomic and molecular studies have identified common driver mutations in well-differentiated thyroid cancer (WDTC) (6-18). *BRAF*^V600E^ and *RAS* mutations are mutually exclusive and represent the most common alterations in WDTC (6-18). WDTC and ATC share driver mutations and some high-risk mutations, including *TERT* promotor and *TP53* mutations. Despite identification of high-risk mutations, clear mechanistic understanding of disease progression, invasion, and metastasis remains limited. Analysis of stromal recruitment and signaling pathway activation is needed to better understand factors that support and enhance thyroid cancer invasion and metastasis.

Wnt signaling upregulation is consistently seen in ATC across molecular subtypes (19, 20). The Wnt/β-catenin pathway is a conserved, developmental signaling pathway that is harnessed to drive many cancers (21-25). Wnt signaling regulates many cellular processes, including cell fate determination, motility, polarity, and stem cell renewal (26). A key step in Wnt signaling is the formation of the Wnt signalosome (25). The Wnt signalosome is a ligand-activated receptor complex that includes the Wnt coreceptors, Frizzled and LRP6, and the cytoplasmic protein, Dishevelled (25). Formation of the Wnt signalosome is required for receptor-mediated stabilization of the transcriptional coactivator, β-catenin. Consequently, β-catenin accumulates in the cytoplasm, enters the nucleus, and binds the TCF/Lef1 family of transcription factors to mediate a Wnt-specific transcriptional program. Several cancers, including colorectal cancer, are known to have Wnt signaling pathway mutations that impair β-catenin degradation or mutations in proteins that promote Wnt signalosome formation (27-29). Other cancers are driven by increases in Wnt ligands themselves (30-32). Within thyroid cancer, several components of the Wnt signaling pathway are known to be altered (19, 33-36).

We recently discovered that ATCs exhibit dramatically high levels of Wnt ligand expression yet few Wnt pathway-activating mutations (10, 19). This result suggests that Wnt signaling may play an important role in the pathophysiology of ATCs. One protein that has been shown to amplify ligand-driven Wnt signaling in development is tenascin-C (TNC). TNC synthesis is tightly regulated in humans, having widespread expression in embryonic tissues and restricted distribution in adult tissues (37, 38). Several studies have proposed the presence of a TNC-Wnt crosstalk during development (39-41). As TNC is a secreted glycoprotein, it may interact with extracellular mediators of Wnt signaling, including Wnt ligands, LRP6, or Frizzled. Studies in the whisker stem cell niche indicate that TNC can bind and concentrate Wnt-3a ligands to upregulate Wnt activation (40). In support of this proposed mechanism, studies of acute kidney injury demonstrated that TNC coimmunoprecipitates with Wnt-1 and Wnt-4 overexpressed in a human kidney cell line (HKC-8) (39). TNC expression in cancer has been proposed to promote invasion and metastasis and is commonly thought to be derived from the tumor stroma (37, 41-47). However, our understanding of TNC expression and TNC-Wnt crosstalk in thyroid cancers is limited.

Here, we investigated the expression of TNC in thyroid cancer and its role in amplifying ligand-driven Wnt signaling. In this study, we use patient sequencing data from 3 large multi-institutional thyroid patient cohorts (9, 10, 48). We demonstrate that *TNC* expression is upregulated in thyroid cancer cells along the tumor's invasive edge and within intravascular spaces. Using coculture and in vitro modeling, we demonstrate an interaction between Wnt ligand and TNC that potentiates Wnt signaling. Finally, in an ATC tumor model, we demonstrate that TNC increases Wnt pathway activation, tumor burden, tumor cell invasion, and metastasis. The interaction between TNC and Wnt is likely integral to thyroid cancer behavior and serves as a potential marker both for prognostication and targeted therapeutics.

## Materials and Methods

This research study was approved by the Vanderbilt University Medical Center Institutional Review Board.

### RNA Sequencing Analysis of Publicly Available Well-Differentiated Thyroid Cancer Data From the Cancer Genome Atlas and Genotype-Tissue Expression Program

GEPIA is a web-based tool that delivers rapid and customizable functions for analyzing The Cancer Genome Atlas (TCGA) and Genotype-Tissue Expression Program (GTEx) data (http://gepia.cancer-pku.cn/) (9, 49). GEPIA was used to compare TNC expression between tumor and normal samples from TCGA and GTEx. GEPIA statistical tests were performed via one-way analysis of variance with a cutoff of *P* less than .01. For additional analyses, TCGA Bulk RNA sequencing and clinical data were downloaded from cBioPortal (cbioportal.org) (50-52). The clinical data included the *BRAF*-like or *RAS*-like designation, whether the patient had lymph nodes positive for the disease at resection of the sequenced primary tumor, and the degree of extrathyroidal extension at resection (none, minimal, or moderate/advanced). TNC expression levels were log_2_-transformed and compared between TCGA tumors with *BRAF*-like vs *RAS*-like phenotypes, tumors with and without associated lymph node metastases, and tumors with no extrathyroidal extension, minimal extrathyroidal extension, or moderate/advanced extrathyroidal extension. Statistical differences in log_2_ TNC expression between groups were calculated using the Wilcoxon rank sum test with Bonferroni correction and plotted using R package ggplot2 3.5.0 (53).

### Bulk RNA Sequencing Analysis of Vanderbilt University Medical Center and University of Washington Patient Cohort

TNC expression was analyzed in a previously published bulk RNA sequencing cohort of 312 thyroid resection specimens (251 patients) from Vanderbilt University Medical Center and University of Washington (VUMC/UW) (10). Samples were grouped into benign (multinodular goiters, follicular adenomas, Hürthle cell adenomas), WDTCs (papillary thyroid carcinomas, follicular-variant papillary thyroid carcinomas, follicular thyroid carcinomas, Hürthle cell carcinomas), poorly differentiated thyroid carcinomas (PDTCs), and ATC. WDTCs were further split into *BRAF*-like or *RAS*-like, as previously described (9, 10). TNC expression levels were log_2_-transformed and compared across diagnoses (benign, WDTC, ATC) and between *BRAF*-like or *RAS*-like WDTCs. Within the entire malignant cohort (WDTC, PDTC, ATC) and the WDTC cohort, the expression of TNC in primary tumors was compared based on the presence or absence of associated lymph nodes and distant metastases. TNC expression was also compared between primary samples and lymph node samples. Statistical significance was calculated using Wilcoxon rank sum tests, and box plots were generated with R package ggplot2 3.5.0 (53). Progression-free survival (PFS) metrics were calculated as previously described for this cohort, using a 50th percentile TNC expression cutoff within all malignant (WDTC, PDTC, and ATC) or WDTC only (10). R packages survival 3.5 to 5 and survminer 0.4.9 were used to generate PFS plots, and statistical significance was determined via log-rank test.

### Bulk RNA Sequencing Analysis of Data From Lee et al Patient Cohort

Raw count matrices and sample meta data for 16 ATCs, 348 PTCs, and 263 normal thyroids from Lee et al (48) were downloaded from Gene Expression Omnibus (https://www.ncbi.nlm.nih.gov/geo/) using accession number GSE213647. Raw counts were TPM-normalized and log_2_-transformed. TNC expression was compared for log2 TPM counts across diagnoses (ATC, PTC, normal thyroid) using the Wilcoxon rank sum test with Bonferroni correction and plotted using R package ggplot2 3.5.0 (53).

### Hallmark Wnt–β-Catenin Score Calculation in Bulk RNA Sequencing Data

For all sequencing cohorts, hallmark Wnt–β-catenin scores were calculated from TPM (Lee, VUMC/UW) or RSEM (TCGA) counts using the R package GSVA 1.48.3 with the Molecular Signatures Database hallmark Wnt–β-catenin signaling gene set and default arguments for the GSVA function (54, 55).

### Cancer-Associated Fibroblast Deconvolution in Bulk RNA Sequencing Data

Estimated cancer-associated fibroblast (CAF) levels for the VUMC/UW bulk RNA sequencing data were previously calculated using the CAF EPIC deconvolution algorithm within TIMER 2.0 (10, 56, 57).

### Bulk RNA Sequencing Data Analyzed With Spearman Correlation

Across the TCGA, Lee et al, and VUMC/UW cohorts, Spearman correlations between Wnt ligands, TNC, Hallmark Wnt signaling, and CAF abundance were calculated and plotted with R packages ggplot2 3.5.0, and corrplot 0.92 (53).

### Multiplex Immunofluorescence of Formalin-fixed Paraffin-embedded Tissue

Multiplex immunofluorescence (IF) was performed as previously described (10). Primary antibodies (Abcam ab207178 recombinant rabbit monoclonal anti-fibroblast activation protein α [FAP] immunoglobulin G [IgG], clone EPR20021, 1:100; Abcam ab88280 mouse monoclonal [EB2] anti-Tenascin-c 1:100) were diluted in blocking buffer and incubated on tissue sections at 4 °C for 16 hours (Abcam; Bioss USA). Tissue sections were washed with 0.05% Tween 20 in phosphate-buffered saline (PBS). Secondary antibodies (Invitrogen A-21245 polyclonal goat anti-rabbit IgG Alexa Fluor 647 1:150; Abcam ab97035 polyclonal goat anti-mouse IgG H&L [Cy3] preadsorbed 1:100) were diluted in blocking buffer containing Hoechst 33342 nuclear stain (1:1000) and incubated on tissue sections at 37 °C for 1 hour (Abcam; Thermo Fisher). Representative 20× images were taken of each tissue section on a Nikon Spinning Disc confocal microscope.

### RNA In Situ Hybridization

Tissue sections (5 µm thickness) were cut from formalin-fixed paraffin-embedded (FFPE) blocks and stored at −20 °C. RNAscope probes Hs-TNC-C1, Hs-WNT2-C1, and Hs-FAP-C2 and RNAScope 2.5 HD Duplex and RNAscope Wash Buffer Reagents were purchased from Advanced Cell Diagnostics. RNAscope was performed according to the manufacturer's guidelines.

### Cell Culture

K1 cells were obtained from Sigma Aldrich. The ATC cell line, THJ-16T, was obtained from Dr John Copland (Mayo Clinic). WPMY-1 and HEK293 (CRL-1573) cells were obtained from American Type Culture Collection (ATCC). Cells were authenticated using STRS analysis and maintained and used experimentally at fewer than 20 passages from thaw. K1 cells were grown in RPMI (VWR) containing 10% fetal bovine serum (ThermoFisher Scientific), 1% penicillin-streptomycin (100 units/mL penicillin and 0.1 mg/mL streptomycin, Sigma), 1X MEM Non-Essential Amino Acids (VWR), and 1 mM sodium pyruvate (Vanderbilt Molecular Biology Resource). WPMY −1 and HEK293 cells were grown in high-glucose Dulbecco’s modified Eagle’s medium (Sigma) containing 8% to 10% fetal bovine serum (ThermoFisher Scientific), and 1% penicillin-streptomycin (100 units/mL penicillin and 0.1 mg/mL streptomycin, Sigma). All cell lines tested negative for mycoplasma contamination.

### Generating 7TFP or TOPFLASH-Stable Cell Lines

Stable Wnt reporter cell lines were generated using lentiviral transduction. Viral media were collected from HEK293FT cells transfected with the 7TFP lentiviral plasmid (Addgene No. 24308), along with the PAX2 (packaging) and pMD2G (envelope) plasmids. Thyroid cancer cell lines were cultured in lentiviral media with 8-mg/mL polybrene for 24 hours. Antibiotic selection was performed with puromycin (10 µg/mL) (Mediatech/CellGro-Corning [MT61385RA]).

### Transfections

Plasmid transfections were performed using Lipofectamine 3000 Transfection reagents (Invitrogen). For cell-based luciferase TOPFLASH assays, K1 cells were overexpressed with either large-splice variant TNC (Addgene 65414) or small-splice variant TNC (Addgene 65415), THJ-16T cells were overexpressed with small-splice variant TNC (Addgene 65415), and WPMY-1 cells were overexpressed with Wnt-2 (Addgene 43809). All flag plasmids were manufactured by Gene Universal. Coimmunoprecipitation plasmid transfections were performed using the calcium phosphate method.

### XAV939 Treatment

After transfection of TNC and Wnt-2, cells were plated in 96-well plates and cocultured for 16 hours before 500 nM of XAV939 (Selleck Chemicals) was added to cells for 24 hours.

### TOPFLASH Assay

Twenty-four hours after transfection alone or transfection followed by XAV939 treatment, cells were lysed as per the CellTiter-Glo 3D Assay (Promega) protocol to quantify cell viability. Briefly, an equal volume of CellTiter-Glo was added to the cell plate and contents were mixed on an orbital shaker for 15 minutes at 150 rpm. The plate was then incubated at room temperature for 10 minutes to allow the luminescent signal to stabilize. One Glo Luciferase Assay (Promega) was performed by adding an equal volume of One Glo to the cells followed by cell lysis for 15 minutes at room temperature. Luminescence and luciferase signals were then quantified using a Synergy NEO (BioTek multi-mode plate reader). Luciferase signals were normalized to cell viability signals using the CellTiter-Glo assay. One-way analysis of variance was performed with Tukey test correction.

### Coimmunoprecipitation Sample Preparation for Cell Lysates

Cells were lysed using nondenaturing lysis buffer (NDLB) (50 mM Tris-HCl pH 7.4, 300 mM NaCl, 5 mM EDTA, and 1% Triton X-100 [w/v]), supplemented with 1 mM PMSF and PhosSTOP phosphatase inhibitor cocktail tablets (Roche). Samples were incubated at 4 °C for 30 minutes, followed by clarification by spinning in a microfuge at 13 000 rpm for 10 min at 4 °C. Lysates were diluted to 1 mg/mL with NDLB and incubated with antibodies with overnight rotation at 4 °C. Samples were then incubated with Protein A/G magnetic beads (Millipore) for 2 hours with rotation at 4 °C. Beads were washed 5 times with NDLB, and sample buffer was added to elute the bound protein (95 °C for 10 minutes). Proteins were analyzed by sodium dodecyl sulfate–polyacrylamide gel electrophoresis and immunoblotting. Fluorescence signal was detected using an Odyssey (LI-COR). Obtained images and band intensity were analyzed using Empiria (LI-COR).

### Coimmunoprecipitation Sample Preparation for Recombinant Proteins

Recombinant TNC (1 µg, EMD Millipore) and recombinant Wnt-2 (Biomatik) were diluted to a total volume of 1 mL with NDLB and incubated with TNC antibody overnight at 4 °C. Samples were then incubated with Protein A/G magnetic beads (Millipore). Beads were washed 5 times with NDLB, and sample buffer was added to elute the bound protein (20 °C for 1 hour). Proteins were analyzed by sodium dodecyl sulfate–polyacrylamide gel electrophoresis and immunoblotting. Chemiluminescence signal was detected using an Odyssey (LI-COR). Obtained images and band intensity were analyzed using Image Studio (LI-COR).

### Antibodies for Immunoblotting

The following antibodies were used for immunoblotting: rabbit anti-FLAG (Proteintech, 20543-1-AP, RRID:AB_11232216), mouse anti-FLAG (Vanderbilt Protein and Antibody Resource, RRID: AB_3674853), mouse anti-tubulin (Developmental Studies Hybridoma Bank, E7, RRID: AB_528499), mouse anti-tenascin C (Abcam, ab3970, RRID:AB_304196), rat anti-tenascin C (R&D Systems, MAB2138-SP, RRID: AB_2203818), rabbit anti-Wnt-2 (Abcam, ab109222, RRID: AB_10862500), rabbit anti-V5 (Cell Signaling, 13202S, RRID:AB_2687461), goat anti-rat IgG H + L-HRP (Thermo Fisher, 31470, RRID: AB_228356), goat anti-mouse IgG H + L-HRP (Promega, W4021, RRID: AB_430834), goat anti-rabbit IgG H + L-HRP (Promega, W4011, RRID: AB_430833), goat anti-rabbit 800 (Licor, RRID: AB_2651127), donkey anti-mouse 800 (Licor, RRID: AB_2716622), goat anti-rabbit 680 (Licor, RRID: AB_2721181), and goat anti-mouse 680 (Licor, RRID: AB_2651128). All primary antibodies were used at 1:1000 dilution except anti-tubulin (1:10 000) and anti-flag (1:2000). All secondary horseradish peroxidase (HRP) antibodies were used at 1:2000 dilution, and all fluorescence antibodies were used at 1:20 000 dilution.

### Mouse Experiments

All procedures were approved by the Institutional Animal Care and Use Committee prior to completion. NOD.PrkdcscidIl2rg^−/−^ (NSG-Jackson Laboratories) were injected with 1 × 10^6^ xenograft THJ-16T cells subcutaneously in the flank using a 25-G SubQ needle affixed to a 1-mL syringe. When tumors became palpable (approximately 1-week post injection), intratumoral injections were performed with 1× PBS (Corning) or recombinant Tenascin-C (EMD Millipore, 0.2 mg/mL) twice weekly. Tumors were measured twice weekly using digital calipers, and mice were weighed weekly to ensure that weight loss did not exceed 20% of body weight. When tumors reached 2 cm in any dimension or ulcerated, mice were humanely euthanized. Male (14 animals) and female (11 animals) mice were used in all experiments, and this was not considered a factor in the statistical analysis.

### Mouse Tumor Histology and Immunohistochemistry

Tumors were resected and weighed, fixed in 10% neutral buffered formalin, processed, and embedded in paraffin. Five µm sections were cut and either stained with hematoxylin and eosin or used for immunohistochemistry (IHC). Slides used for IHC were stained by the Translational Shared Pathology Resource (TPSR) center. Slides were placed on a Leica Bond Max IHC stainer. Heat-induced antigen retrieval was performed using Epitope Retrieval 2 solution for 20 minutes. Slides were placed in a Protein Block (reference No. x0909, DAKO) for 10 minutes. Slides were incubated with anti–β-catenin (Cell Signaling, 9582) for 1 hour at a 1:100 dilution. The Bond Polymer Refine detection system was used for visualization. Slides were the dehydrated, cleared, and cover-slipped.

### Statistics

Tumor volumes and tumor weights from 3 experiments (batches) were analyzed. A linear mixed-effects model was used to evaluate the association between treatment and Wnt reporter activation and account for correction due to technical replicates from the same biological samples. Wnt reporter activation by treatment group was estimated using least-squares means (ie, model-based means), and differences among groups were compared using the Wald test with Tukey test correction. We employed the power variance function to address heteroscedasticity across treatment groups. Batch was included as a covariate to adjust for potential batch effects. Residual analysis was conducted to verify the model's assumptions. Tumor volumes were log-transformed (with the addition of 1 to avoid log(0)) to address heterogeneity detected in residual analysis. Statistical analyses were performed in R v4.4.0.

## Results

### Tenascin-C Expression Is Increased in Well-Differentiated Thyroid Cancer and Anaplastic Thyroid Cancer

**Figure 1.**
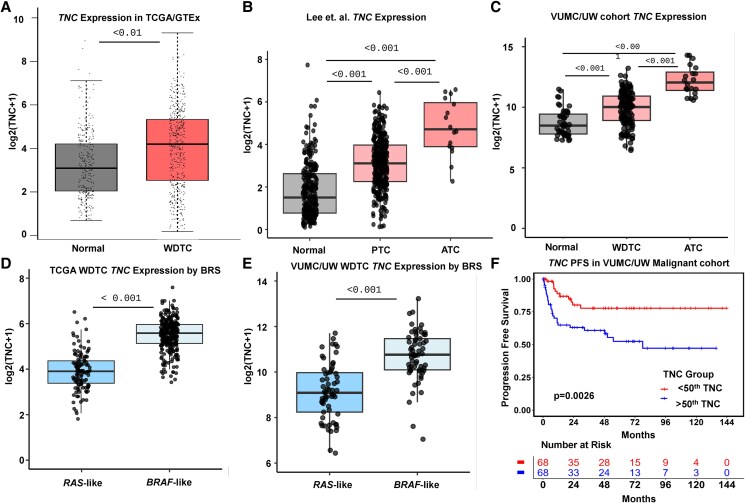
Tenascin-C (TNC) expression is higher in thyroid cancer patient samples. A, Box plots generated with GEPIA showing TNC expression in TCGA and GTEx thyroid samples split by normal thyroid vs well-differentiated thyroid cancer (WDTC). B and C, Box plots showing *TNC* expression in B, Lee et al showing normal, papillary thyroid carcinoma (PTC), and anaplastic thyroid cancer (ATC) samples and C, primary samples by diagnosis (benign = n of 46; WDTC = n of 136; ATC = n of 20). *P* values calculated using Wilcoxon rank sum test with Bonferroni correction. D and E, *TNC* expression based on *RAS*-like or *BRAF*-like gene expression phenotypes in D, TCGA WDTC Cell 2014 cohort and E, VUMC/UW primary WDTCs. *P* values calculated using Wilcoxon rank sum test with Bonferroni correction. F, Progression-free survival (PFS) plot for all malignant patients with less than 50th percentile *TNC* expression or greater than 50th percentile TNC expression in primary tumors. *P* values calculated using log-rank test.

To first analyze TNC expression in thyroid cancer, we assessed TCGA WDTC (n = 512) and GTEx normal thyroid (n = 337) gene expression using GEPIA. *TNC* expression was upregulated in WDTC compared to normal thyroid tissue (Fig. 1A;  *P* < .01). Additionally, *TNC* is known to have several different splicing events, so we evaluated the different isoforms of TNC present in thyroid cancer (58). TNC isoforms 12, 11, and 1 are the most common (Supplementary Fig. S1A and S1B) (59). We next investigated *TNC* expression in the more aggressive thyroid cancer, ATC. Using publicly available sequencing data from Lee et al, we assessed *TNC* expression in normal, PTC (the most common type of WDTC), and ATC samples. We found that *TNC* expression is increased in PTC relative to normal (*P* < .001) and ATC relative to normal (*P* < .001), with the highest expression in ATC (Fig. 1B).

We recently collected and published an analysis of a large cohort of whole-exome and bulk RNA sequencing data analysis of benign and malignant thyroid tissue (10). This cohort was enriched for ATC and WDTCs with metastases and poor outcomes to improve detection of drivers of aggressive disease. Similar to the cohort from Lee et al, *TNC* expression is increased in WDTC (*P* < .001) and ATC samples (*P* < .001), with the highest increase in ATC (Fig. 1C). We further classified WDTCs into *RAS*-like or *BRAF*-like based on their gene expression profiles (9, 10). Using the TCGA WDTC cohort, *TNC* expression is increased in *BRAF*-like WDTCs compared to *RAS*-like tumors (Fig. 1D;  *P* < .001). Similar to TCGA WDTCs, we observed a statistically significant increase in our cohort in *TNC* expression within WDTCs that are *BRAF*-like compared to *RAS*-like (Fig. 1E;  *P* < .001). Taken together, *TNC* expression is upregulated in thyroid cancer compared to normal tissue. The highest increases in *TNC* are observed in *BRAF*-like WDTCs and ATCs. Finally, using PFS analysis of our whole malignant cohort, we see an improved survival in patients with low *TNC* expression (split by 50th percentile, Fig. 1F;  *P* = .0026). This statistical significance is not seen within our WDTC cohort (Supplementary Fig. S1C; *P* = .46) (59), suggesting that this survival difference is likely driven, at least in large part, by the high *TNC* expression in lethal ATCs.

### Tenascin-C Expression Is Increased in Metastasis

**Figure 2.**
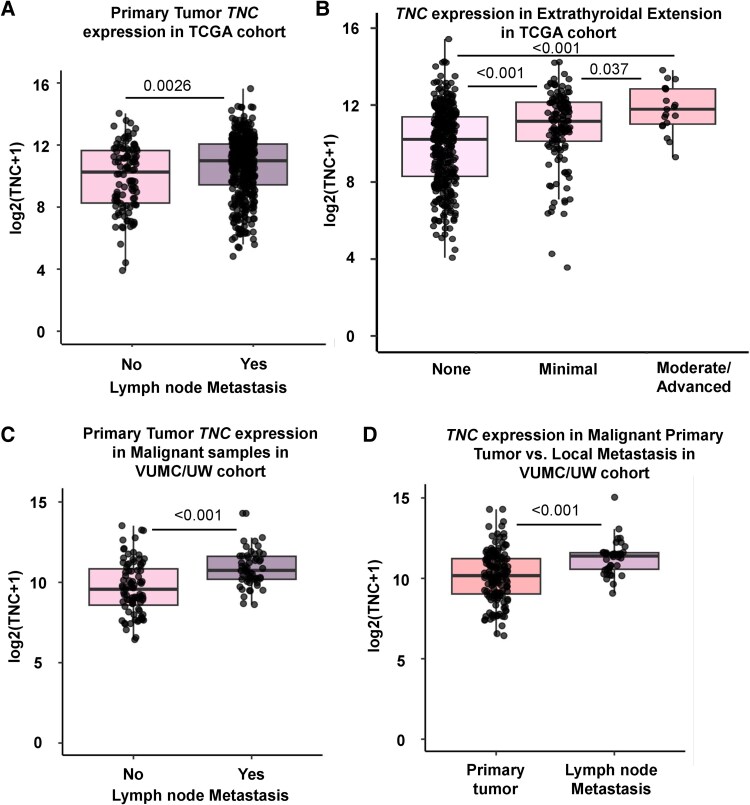
Tenascin-C (TNC) expression is correlated with local invasion and lymph node metastasis. A and B, Box plots showing *TNC* expression in TCGA WDTCs split by A, presence (YES) or absence (NO) of lymph node spread and B, extent of extrathyroidal extension (none, minimal, moderate/advanced). *P* values calculated using Wilcoxon rank sum test with Bonferroni correction. C, Box plots showing *TNC* expression in primary malignant tumors from the VUMC/UW cohort split by presence (YES) or absence (NO) of associated local metastases. D, Box plots showing *TNC* expression in primary malignant tumors vs lymph node metastases in the VUMC/UW cohort. *P* values calculated using Wilcoxon rank sum test with Bonferroni correction.

TNC expression has been implicated as a driver of metastasis, so we further assessed the association between *TNC* and metastasis in our thyroid cancer cohorts (60). Within the TCGA WDTC cohort, primary tumors from patients with lymph node metastases showed higher overall *TNC* expression compared to primary tumors from patients without lymph node metastases (Fig. 2A; *P* = .0026) (9). Additionally, *TNC* expression was increased in tumors with minimal (*P* < .001) and moderate/advanced (*P* < .001) extrathyroidal extension (Fig. 2B). An evaluation of TNC and metastasis within the VUMC/UW cohort shows similar findings. *TNC* expression is increased in the primary tumors of patients with lymph node metastases (Fig. 2C; *P* < .001) when evaluating our whole malignant cohort as well as when we restricted the cohort to only WDTC (Supplementary Fig. S2A; *P* < .001) (59). This analysis confirms that our correlation is not driven solely by ATC. While TNC expression in the primary tumors also correlates with distant metastatic disease, this trend is not statistically significant (Supplementary Fig. S2B and S2C) (59). Interestingly, not only is TNC increased within the primary tumors of patients with lymph node metastases, but it is also increased within the metastatic cells of the lymph nodes (Fig. 2E;  *P* < .001 and Supplementary Fig. S2D, *P* < .001) (59). In conclusion, across multiple published thyroid cancer sequencing cohorts, we observe an increase in TNC in malignant samples. This increase is highest in samples with aggressive behavior, including lymph node metastases, extrathyroidal extension, and transformation to ATC.

### Spatial Localization of RNA and Protein Expression of Tenascin-C in Anaplastic Thyroid Cancer

**Figure 3.**
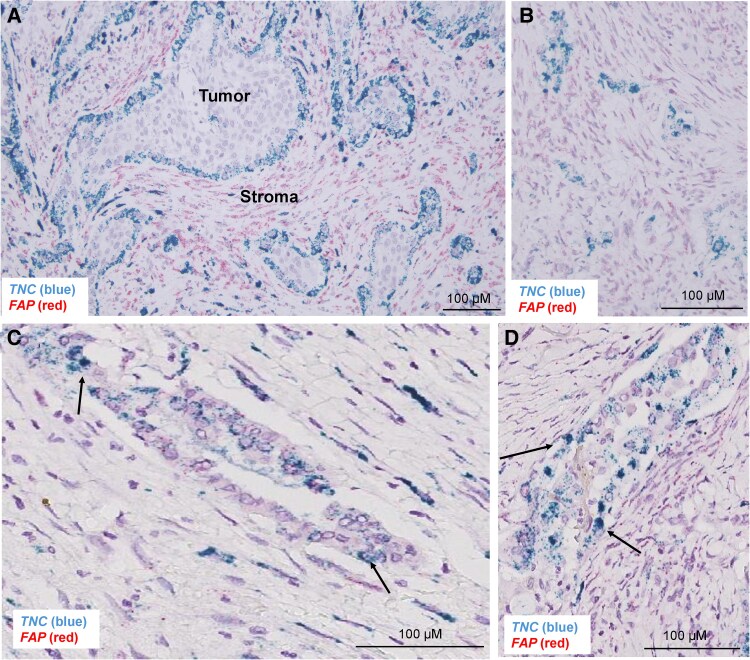
Tenascin-C (TNC) expression is along the epithelial-stromal border in anaplastic thyroid cancer (ATC). A and B, RNAscope of representative ATC samples showing staining for *TNC* or fibroblast-activating protein (*FAP*). C and D, RNAscope of representative papillary thyroid carcinoma (PTC) regions within ATC samples showing staining for *TNC* or *FAP*.

While it is commonly thought that TNC is expressed by fibroblasts in the tumor microenvironment (44, 45), recent studies also suggest that tumor cells both in breast and head and neck cancer can produce TNC (46, 47). To identify which cells are making TNC in thyroid cancer and their spatial localization, we performed RNA in situ hybridization and multiplex IF on patient ATCs. First, using RNA in situ hybridization, we probed for *TNC* and *FAP*, a ubiquitous CAF marker. We found that 7 of 12 samples (58%) have *TNC* staining of tumor cells along the invasive edge (Fig. 3A and 3B). In addition, 10 of 12 samples (83%) have *TNC* staining of endothelial cells and tumor cells within blood vessels (intravascular invasion; Fig. 3C and 3D). Finally, all 12 samples (100%) showed fibroblast *TNC* staining (see Fig. 3A and 3B).

**Figure 4.**
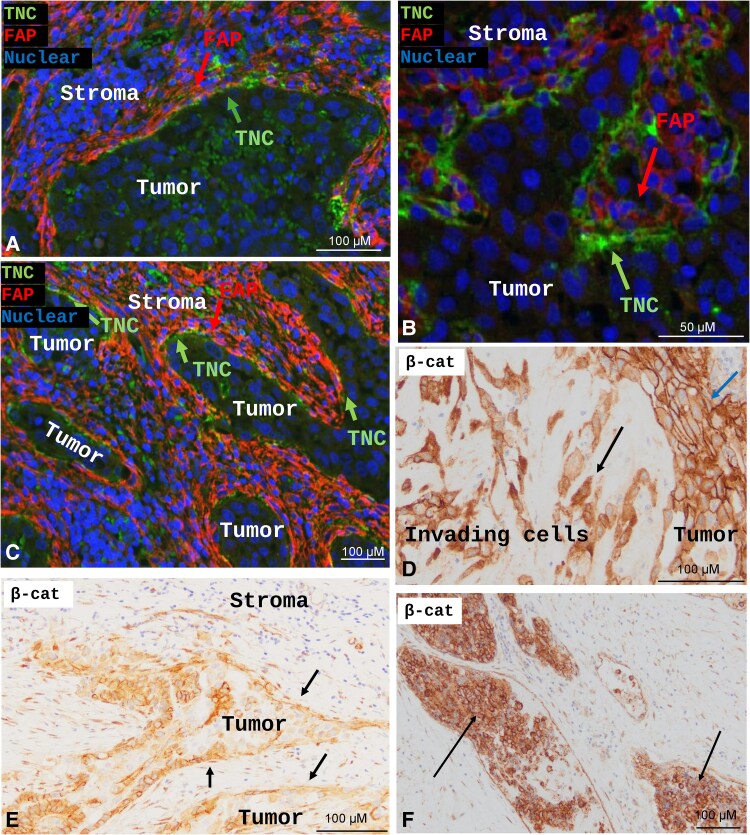
β-Catenin and tenascin-C (TNC) protein expression confirms RNA expression. A to C, Multiplex-immunofluorescence staining of anaplastic thyroid cancer (ATC) patient samples showing TNC staining, fibroblast-activating protein (FAP), and Hoechst nuclear stain. D to F, Immunohistochemistry of β-catenin from ATC patient tumors.

To further confirm protein-level expression of TNC and enhanced Wnt signaling in our patient tissues, we used multiplex IF and IHC of FFPE patient surgical tissues. We first confirmed TNC protein expression using multiplex IF staining and showed TNC protein expression along the tumor-stroma border and in areas of invading tumor cells (Fig. 4A-4C). This protein expression overlayed with areas of increased TNC RNA, identified in our RNAScope analysis. Additionally, given previous publications from our group showing that ATCs are characterized by increased canonical Wnt signaling (19), we investigated nuclear β-catenin IHC in these patient tissues. ATC patient tissues showed increased cytoplasmic and nuclear β-catenin staining within the TNC-expressing cells that are at the tumor-stroma border, are invading into the surrounding stroma, or are located within blood vessels (Fig. 4D-4F). Adjacent tumor cells that lack TNC expression have predominantly membranous β-catenin. Altogether, we confirm our messenger RNA expression findings by identifying protein expression of TNC along the tumor-stroma border and in areas of invasion. We also show that these same TNC-expressing tumor cells have activated Wnt signaling, as demonstrated by nuclear translocation of β-catenin.

### Tenascin-C and Wnt-2 Expression Correlate in Thyroid Cancer

**Figure 5.**
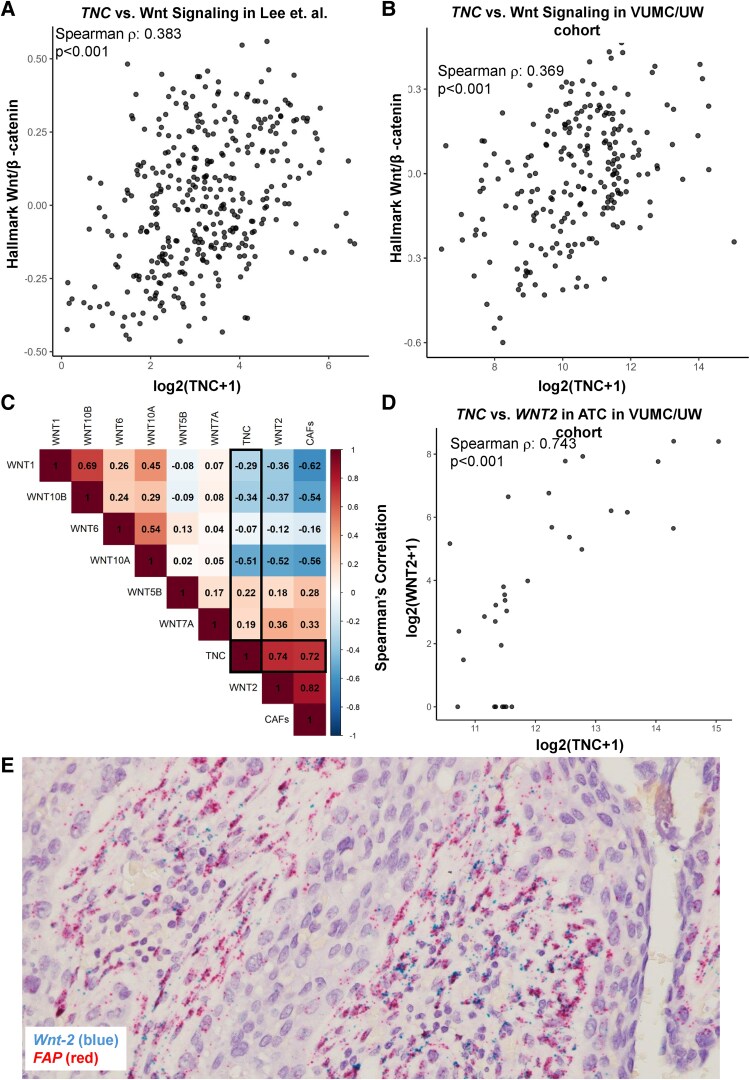
Tenascin-C (TNC) and Wnt-2 expression are correlated in anaplastic thyroid cancer (ATC). A and B, Spearman correlation between hallmark Wnt/β-catenin gene activity in A, Lee et al or B, VUMC/UW bulk RNA-sequencing cohorts for all malignant samples. C, Plot of Spearman correlations between Wnt ligands, cancer-associated fibroblasts (CAFs), and *TNC* in VUMC/UW ATCs. D, Spearman correlation between TNC and *WNT2* in VUMC/UW ATC samples. E, RNAscope of representative ATC sample showing staining for *WNT2* or fibroblast-activating protein (*FAP*).

Although TNC is found at the invasive border of ATC, the mechanism by which it influences tumor invasion is unclear (46, 47). In nonneoplastic disease, TNC has been shown to interact with Wnt ligands and augment ligand-dependent Wnt signaling (39, 41). As aggressive thyroid tumors have increased expression of Wnt ligands, we hypothesized that TNC acts to enhance Wnt signaling at the invasive border of thyroid tumors through interactions with Wnt ligands. To investigate the relationship between TNC and Wnt signaling, we first looked at hallmark Wnt/β-catenin signaling gene activity and observed a positive correlation with *TNC* expression in all malignant samples using the Lee et al cohort (Fig. 5A; *P* < .001) and our VUMC/UW cohort (Fig. 5B; *P* < .001). We observed the same positive correlation when we looked at WDTC *TNC* expression and Hallmark Wnt/β-catenin signaling gene activity across all patient cohorts (Supplementary Fig. S3A-S3C) (59).

Next, we aimed to determine if there are specific Wnt ligands that TNC is interacting with to potentiate Wnt signaling. We recently identified 7 Wnt ligands (Wnt-1, Wnt-2, Wnt-5b, Wnt-6, Wnt-7a, Wnt-10a, Wnt-10b) that are upregulated in ATC (19). Because we spatially observed TNC next to fibroblasts, we chose to look at the correlation between TNC, ATC-upregulated Wnt ligands, and CAF abundance in ATCs (Fig. 5C). We identified a markedly stronger correlation between *TNC* and *WNT2* than any other Wnt ligand (Fig. 5C and 5D; ρ = 0.74; *P* < .001). Additionally, both *TNC* and *WNT2* were correlated with CAFs (ρ = 0.72 and 0.82, respectively). We confirmed the association between *TNC* and *WNT2* across malignant and WDTC-restricted cohorts from Lee et al, TCGA, and VUMC/UW (Supplementary Fig. S3D-S3H) (59). As multiple reports have indicated that Wnt-2 is expressed by CAFs, we anticipated that Wnt-2 may be interacting with TNC via CAFs adjacent to invasive, TNC-expressing tumor cells (61-63). To identify whether Wnt-2 is produced in close proximity to the invasive border of tumor cells, we performed RNA in situ hybridization, probing for *WNT2* and *FAP* within ATC tumors. We found that *WNT2* is made by CAFs within the tumor microenvironment adjacent to the leading edge of tumor cells (Fig. 5E). In conclusion, across patient cohorts, TNC is correlated with hallmark Wnt/β-catenin gene activity. Among Wnt ligands, TNC exhibits the strongest correlation with Wnt-2, which is produced by CAFs at the invasive border with tumor cells, suggesting a possible interaction between Wnt-2 and TNC.

### Tenascin-C Potentiates Wnt Signaling and Directly Interacts With Wnt-2

**Figure 6.**
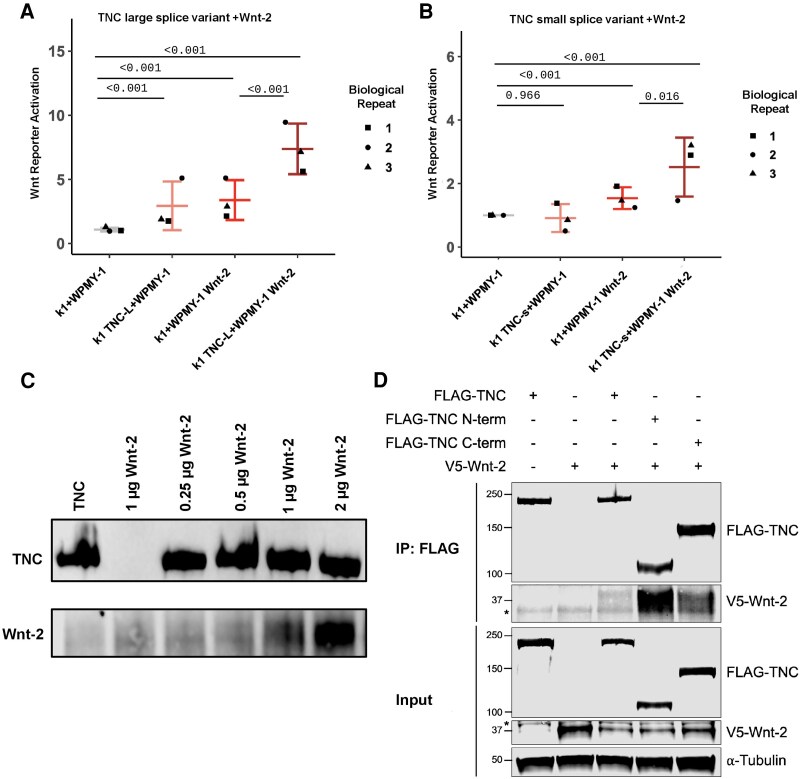
Tenascin-C (TNC) variants potentiate Wnt signaling in the presence of Wnt ligands. A and B, TOPFLASH Wnt reporter readouts for K1 cells overexpressing A, TNC large-splice variant cocultured with Wnt-2 expressing WPMYs or B, TNC small-splice variant cocultured with Wnt-2 expressing WPMYs. *P* values were calculated using Wald test with Tukey test correction, (K1 + WPMY-1) (K1 TNC variant + WPMY-1), (K1 + WPMY-1 Wnt-2), and (K1 TNC variant + WPMY-1 Wnt-2), and each biological replicate is represented as a different shape. C, TNC and Wnt-2 coimmunoprecipitation using several concentrations of recombinant Wnt-2 and 0.1 mg/mL of recombinant TNC. D, Flag-tagged TNC and V5-tagged Wnt-2 co-overexpressed in HEK293 cells was immunoprecipitated (IP) with anti-FLAG antibody and protein A/G beads, and coimmunoprecipitated V5-Wnt2 was detected by immunoblotting. WCL, whole-cell lysates. *Nonspecific band.

TNC has been shown to influence Wnt signaling by interacting directly with Wnt ligands (39, 41). To study the crosstalk between TNC and Wnt ligands in vitro, we used a TOPFLASH K1 thyroid cancer cell line. First, we tested whether TNC could potentiate ligand-driven Wnt signaling using a coculture method. Specifically, we overexpressed TNC in the TOPFLASH K1 thyroid cancer cell line and overexpressed Wnt-2 in a fibroblast cell line, WPMY. With no overexpression, or with only TNC overexpression, we observe baseline activation of the Wnt signaling pathway. With Wnt-2 overexpression, we see activation of Wnt signaling. However, Wnt signaling is potentiated when we overexpress both TNC and Wnt-2, independent of whether the small or large TNC splice variant is overexpressed (Fig. 6A and 6B). To further confirm this potentiation in an ATC cell line, we used a TOPFLASH THJ-16T ATC cell line and saw similar results (Supplementary Fig. S4A) (59). As expected, this potentiation is abolished in the presence of a Wnt inhibitor, XAV939 (Supplementary Fig. S4B) (59). These data suggest a role for TNC in potentiating Wnt signaling in the tumor microenvironment through Wnt-ligand mediated activation.

To further explore an interaction between TNC and Wnt ligand, we used recombinant TNC and recombinant Wnt-2 and performed coimmunoprecipitation experiments, in which we pulled down TNC and immunoblotted for Wnt-2. Wnt-1, 3a, and 4 have previously been shown to coimmunoprecipitate with TNC, but the interaction between TNC and Wnt-2 has not been previously described (39, 40). We found that Wnt-2 pulled down with recombinant TNC, indicating a potential interaction between the two proteins (Fig. 6C). To explore the interaction between TNC and Wnt-2 further, we FLAG tagged the small-splice variant of TNC and created 2 distinct TNC truncation constructs consisting of either the N-terminus, containing epidermal growth factor–like repeats, or the C-terminus, containing FN3-like repeats (Supplementary Fig. S1B) (59). We then co-overexpressed each construct of TNC with V5-tagged Wnt-2 and performed coimmunoprecipitation experiments. We found that Wnt-2 immunoprecipitated with all 3 TNC constructs, providing further support for the interaction between TNC and Wnt-2. Wnt-2 was pulled down most strongly with the N-terminus of the small-splice variant of TNC, indicating that the Wnt-2 binding site on TNC is likely contained within the N-terminal region (Fig. 6D). Surprisingly, much weaker pulldown was observed with the full-length TNC fragment, suggesting that the binding sites for Wnt ligands on TNC may be partially conformationally masked.

### Tenascin-C Increases Tumor Burden In Vivo

**Figure 7.**
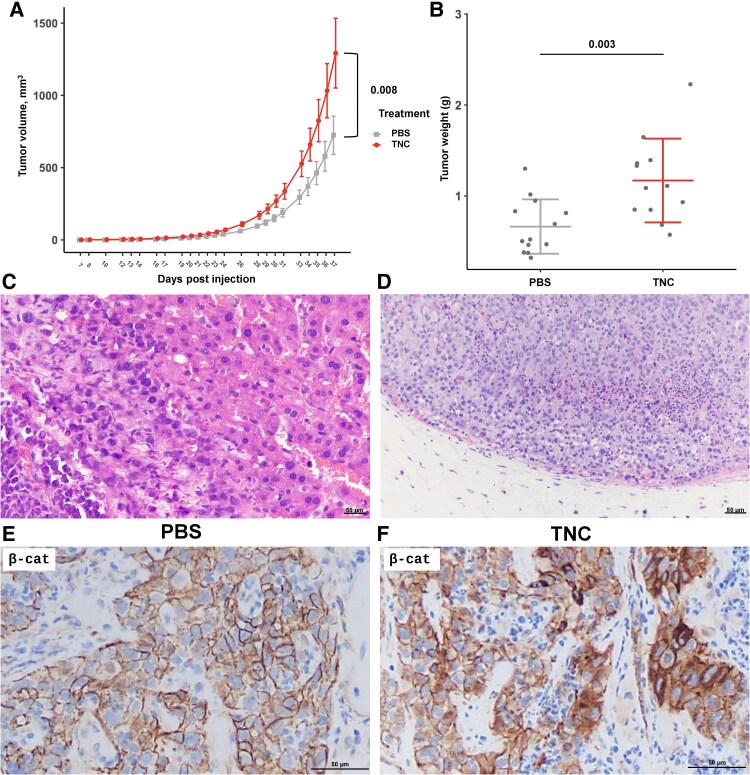
Tenascin-C (TNC) expression increases tumor burden in vivo. A, THJ-16T tumor burden (mm^3^) measured over time for tumors with phosphate-buffered saline (PBS) injections vs tumors with recombinant TNC injections. *P* values calculated using linear mixed-effect model. B, THJ-16T tumor weight of PBS vs TNC injections after resection. *P* values calculated using one-way analysis of variance. C, Hematoxylin-eosin stain (H&E) of THJ-16T tumor invading the liver of TNC-treated mouse. D, H&E of lymph node from TNC-treated mouse that is completely replaced by 16T-PDX tumor. E, Immunohistochemistry of β-catenin from PBS-treated THJ-16T tumors. F, Immunohistochemistry of β-catenin from TNC-treated 16T-PDX tumors.

Finally, we explored the role of TNC in thyroid tumor growth and invasion in vivo. To do so, we injected the THJ-16T patient-derived ATC xenograft cell line subcutaneously into the flanks of NSG mice. THJ-16T forms a similar morphology tumor to the ATC we commonly observe in TNC-positive patient tumors, with nests of squamoid tumor cells surrounded by CAFs (Supplementary Fig. S5A and S5B) (59). After the tumors became palpable, approximately 7 days after injection, either PBS or recombinant TNC was injected twice weekly into the tumors of these mice, and tumor size was measured. We saw a statistically significant increase in tumor size (*P* = .008) and weight (*P* = .003) in our TNC mice compared to our PBS controls (Fig. 7A and 7B). In addition, in 4 TNC-treated mice, we observed dramatic cancer cell invasion that extended into the rib cage, pleura, lung, peritoneum, and liver. Metastatic disease was also present in 3 TNC-treated mice with tumors present in the spleen and lymph nodes (Fig. 7C and 7D; Supplementary Fig. S5C) (59). PBS-treated tumors were found only subcutaneously in the skin of injected mice. After the humane end point was reached, tumors were collected for histologic evaluation and β-catenin IHC. IHC staining demonstrated membranous β-catenin staining with rare cytoplasmic staining in PBS-treated tumors (Fig. 7E). However, strong nuclear and cytoplasmic β-catenin staining was identified in mice treated with recombinant TNC, indicating activation of the Wnt pathway in TNC-treated tumors (Fig. 7F). Taken together, these findings show that TNC treatment led to an increase in Wnt activation, tumor burden, tumor cell invasion, and tumor metastasis.

## Discussion

TNC is expressed widely in development but is largely silenced in adulthood. In this study, using multiple large patient cohorts, in vitro models, and in vivo mouse models, we identified TNC as an important modulator of Wnt signaling in thyroid cancer. Our data demonstrate that TNC expression is tightly regulated in thyroid cancer, as it is expressed by tumor cells along the leading edge and within areas of intravascular invasion. In these same regions along the leading edge and in areas of intravascular invasion, we also confirm an increase in Wnt signaling. The TNC-expressing tumor cells along the leading edge are immediately adjacent to Wnt-2–producing fibroblasts. This close approximation allows for a TNC-Wnt interaction that leads to the potentiation of Wnt signaling within the invading tumor cells. Furthermore, with the addition of a Wnt inhibitor, we abolish this potentiation of Wnt signaling. Based on our in vivo animal studies, we speculate that this potentiation of Wnt signaling by TNC plays an important role in driving thyroid cancer invasion and metastasis. Until now, few drivers of thyroid cancer invasion have been identified. Thus, TNC may then serve as an important marker that could be used to inform both prognosis and treatment.

This study also has broad implications beyond thyroid cancer. TNC had been previously shown to interact with Wnt ligands in a handful of developmental or fibrosis models. We show here that within thyroid cancer there is an interaction between TNC and Wnt-2 that leads to pathway activation. To our knowledge, we are the first to map the interaction between TNC and Wnt-2, and we further explore the mechanism of this interaction. The existence of a novel Wnt modulator could lead to new research directions for cancer therapeutics and beyond. First, TNC is expressed in many tissues and is important in metastasis and several protumorigenic roles. Specifically, in breast cancer, TNC splice variants have been known to increase invasion (64). Further, the mechanism behind TNC's pro-oncogenic effect may be through its interaction with Wnt ligands. Within pancreatic cancer, a crosstalk between TNC and Wnt signaling has already been established (65). The ability of TNC to modulate Wnt signaling across a variety of cancers could lead to the development of target therapies both for TNC and Wnt. Second, Wnt-2 is expressed in many tissues and is important in development and tissue patterning. In the liver, Wnt-2 is expressed by endothelial cells in the central vein and is essential for regulating liver zonation (66). TNC expression has also been identified in inflammatory models of liver disease, fatty liver disease, fibrotic liver disease, and liver transplant rejection (67, 68). The ability of TNC to modulate Wnt signaling across a variety of human diseases could lead to new therapeutic directions for many Wnt-driven disease processes.

There are several important limitations of our study. First, analysis of TNC levels in tumor cohorts is performed using bulk sequencing. Such a sequencing approach limits the ability to detect the specific cell types that express TNC and Wnt-2. To overcome this limitation, we have performed RNAScope and multiplex IF to localize their expression within patient tissues. Another limitation of our study is that both of our TNC variants (N-terminus and C-terminus) include the fibrinogen-like globe domain. As both variants pull down Wnt-2, it is possible that the fibrinogen-like globe domain binds Wnt-2. Additional studies are needed to precisely elucidate the exact site of Wnt-2-TNC interaction. Finally, our in vivo mouse studies use a flank model of ATC. This model was chosen over an orthotopic model since neck ATCs lead to rapid airway compression, limiting our ability to perform studies on tumor growth and metastasis. However, a limitation of the flank model is that the tumor vasculature may not produce the same metastatic disease as an orthotopic model. Despite this limitation, TNC-treated mice with flank tumors showed metastasis to draining lymph nodes and spleen.

## Conclusion

TNC, a hexameric glycoprotein, has been known to increase tumor invasion and metastasis in nonthyroid cancers. Using multiple patient cohorts with RNA sequencing data, we show that thyroid cancer expresses elevated levels of *TNC*. Within local metastasis, ATCs, and *BRAF*-mutant WDTCs, we observe increased *TNC* expression. Using RNA in situ hybridization and multiplex IF, we are able to localize this expression within the tissue. We see a striking pattern emerge with *TNC* expression in a single-cell layer along the epithelial-stromal border and expressed by tumor cells themselves. Additional *TNC* expression is identified at sites of intravascular invasion, directly linking *TNC* expression with metastasis. Mechanistically, we show that both the large- and small-splice variants of TNC bind to Wnt-2 ligand and potentiate Wnt signaling. Finally, in vivo mouse tumors show an increase in tumor size, tumor invasion, metastasis, and Wnt activation when recombinant TNC is injected intratumorally. Taken together, we propose that TNC potentiates Wnt signaling and increases thyroid cancer tumor burden. These findings suggest that TNC may be a useful biomarker of thyroid cancer invasion and metastasis. In addition, targeting TNC may lead to therapeutic advances for some of the most aggressive and lethal thyroid cancers.

## Data Availability

There are restrictions to the availability of patient clinical and sequencing data. This is a retrospective cohort, and it is not possible to consent these patients with historic samples, particularly those with highly aggressive and rapidly lethal disease. As such, the institutional review board (IRB) has requested that we not publicly share individual-level sequencing data from each patient. The data are securely stored within a Vanderbilt patient data system. Aggregate-level data reported in this paper will be shared by the lead contact (V.L.W.) on request. Individual-level data are available only through collaboration following approval of the lead contact and The Vanderbilt University Medical Center IRB. If the lead contact should leave the institution, collaboration requests should be directed to the Pathology Department Chair (Dr Alice Coogan, alice.coogan@vumc.org). Code for all analyses is available at the following link on GitHub: https://github.com/xgj797/Molecular-Signature-Incorporating-Immune-Microenvironment-Enhances-Thyroid-Cancer-Outcome-Prediction, and https://github.com/hartheat/Tenascin-c-potentiates-Wnt-signaling-in-thyroid-cancer.

## Abbreviations

ATC: anaplastic thyroid cancer
CAF: cancer-associated fibroblast
FAP: fibroblast activation protein
FFPE: formalin-fixed paraffin-embedded
GTEx: Genotype-Tissue Expression Program
HRP: horseradish peroxidase
IF: immunofluorescence
IgG: immunoglobulin G
IHC: immunohistochemistry
NDLB: nondenaturing lysis buffer
PBS: phosphate-buffered saline
PDTC: poorly differentiated thyroid carcinoma
PFS: progression-free survival
TCGA: The Cancer Genome Atlas
TNC: tenascin-C
TPSR: Translational Shared Pathology Resource
VUMC/UW: Vanderbilt University Medical Center and University of Washington

## References

[1] Rahib L, Wehner MR, Matrisian LM, Nead KT. Estimated projection of US cancer incidence and death to 2040. JAMA Netw Open. 2021;4(4):e214708. 33825840 10.1001/jamanetworkopen.2021.4708 PMC8027914

[2] Schlumberger MJ . Papillary and follicular thyroid carcinoma. N Engl J Med. 1998;338(5):297‐306. 9445411 10.1056/NEJM199801293380506

[3] Boucai L, Zafereo M, Cabanillas ME. Thyroid cancer: a review. JAMA. 2024;331(5):425‐435. 38319329 10.1001/jama.2023.26348

[4] Califano I, Smulever A, Jerkovich F, Pitoia F. Advances in the management of anaplastic thyroid carcinoma: transforming a life-threatening condition into a potentially treatable disease. Rev Endocr Metab Disord. 2024;25(1):123‐147. 37648897 10.1007/s11154-023-09833-1

[5] Li W, Li Y, Li J, Pang H. Combination of novel therapies and new attempts in anaplastic thyroid cancer. Technol Cancer Res Treat. 2023;22:15330338231169870. 37122242 10.1177/15330338231169870 PMC10134164

[6] Singh A, Ham J, Po JW, Niles N, Roberts T, Lee CS. The genomic landscape of thyroid cancer tumourigenesis and implications for immunotherapy. Cells. 2021;10(5):1082. 34062862 10.3390/cells10051082 PMC8147376

[7] Acuna-Ruiz A, Carrasco-Lopez C, Santisteban P. Genomic and epigenomic profile of thyroid cancer. Best Pract Res Clin Endocrinol Metab. 2023;37(1):101656. 35461756 10.1016/j.beem.2022.101656

[8] Mady LJ, Grimes MC, Khan NI, et al Molecular profile of locally aggressive well differentiated thyroid cancers. Sci Rep. 2020;10(1):8031. 32415114 10.1038/s41598-020-64635-8 PMC7229018

[9] Network CGAR . Integrated genomic characterization of papillary thyroid carcinoma. Cell. 2014;159(3):676‐690. 25417114 10.1016/j.cell.2014.09.050 PMC4243044

[10] Xu GJ, Loberg MA, Gallant JN, et al Molecular signature incorporating the immune microenvironment enhances thyroid cancer outcome prediction. Cell Genom. 2023;3(10):100409. 37868034 10.1016/j.xgen.2023.100409 PMC10589635

[11] Gopal RK, Kubler K, Calvo SE, et al Widespread chromosomal losses and mitochondrial DNA alterations as genetic drivers in hurthle cell carcinoma. Cancer Cell. 2018;34(2):242‐255 e5. 30107175 10.1016/j.ccell.2018.06.013 PMC6121811

[12] Ganly I, Makarov V, Deraje S, et al Integrated genomic analysis of hurthle cell cancer reveals oncogenic drivers, recurrent mitochondrial mutations, and unique chromosomal landscapes. Cancer Cell. 2018;34(2):256‐270.e5. 30107176 10.1016/j.ccell.2018.07.002 PMC6247912

[13] Rivera M, Ricarte-Filho J, Knauf J, et al Molecular genotyping of papillary thyroid carcinoma follicular variant according to its histological subtypes (encapsulated vs infiltrative) reveals distinct BRAF and RAS mutation patterns. Mod Pathol. 2010;23(9):1191‐1200. 20526288 10.1038/modpathol.2010.112 PMC4573468

[14] Jin M, Song DE, Ahn J, et al Genetic profiles of aggressive variants of papillary thyroid carcinomas. Cancers (Basel). 2021;13(4):892. 33672707 10.3390/cancers13040892 PMC7924361

[15] Duan H, Liu X, Ren X, Zhang H, Wu H, Liang Z. Mutation profiles of follicular thyroid tumors by targeted sequencing. Diagn Pathol. 2019;14(1):39. 31077238 10.1186/s13000-019-0817-1 PMC6511182

[16] Yoo SK, Lee S, Kim SJ, et al Comprehensive analysis of the transcriptional and mutational landscape of follicular and papillary thyroid cancers. PLoS Genet. 2016;12(8):e1006239. 27494611 10.1371/journal.pgen.1006239 PMC4975456

[17] Song E, Song DE, Ahn J, et al Genetic profile of advanced thyroid cancers in relation to distant metastasis. Endocr Relat Cancer. 2020;27(5):285‐293. 32163911 10.1530/ERC-19-0452

[18] Yoo SK, Song YS, Lee EK, et al Integrative analysis of genomic and transcriptomic characteristics associated with progression of aggressive thyroid cancer. Nat Commun. 2019;10(1):2764. 31235699 10.1038/s41467-019-10680-5 PMC6591357

[19] Diaz D, Bergdorf K, Loberg MA, et al Wnt/beta-catenin signaling is a therapeutic target in anaplastic thyroid carcinoma. Endocrine. 2024;86(1):114‐118. 38806891 10.1007/s12020-024-03887-0 PMC11444896

[20] Lu L, Wang JR, Henderson YC, et al Anaplastic transformation in thyroid cancer revealed by single-cell transcriptomics. J Clin Invest. 2023;133(11):e169653. 37053016 10.1172/JCI169653 PMC10231997

[21] Garcia-Rostan G, Camp RL, Herrero A, Carcangiu ML, Rimm DL, Tallini G. Beta-catenin dysregulation in thyroid neoplasms: down-regulation, aberrant nuclear expression, and CTNNB1 exon 3 mutations are markers for aggressive tumor phenotypes and poor prognosis. Am J Pathol. 2001;158(3):987‐996. 11238046 10.1016/s0002-9440(10)64045-x PMC1850336

[22] Kurihara T, Ikeda S, Ishizaki Y, et al Immunohistochemical and sequencing analyses of the Wnt signaling components in Japanese anaplastic thyroid cancers. Thyroid. 2004;14(12):1020‐1029. 15650354 10.1089/thy.2004.14.1020

[23] Nagaiah G, Hossain A, Mooney CJ, Parmentier J, Remick SC. Anaplastic thyroid cancer: a review of epidemiology, pathogenesis, and treatment. J Oncol. 2011;2011:542358. 21772843 10.1155/2011/542358 PMC3136148

[24] Zhang Y, Wang X. Targeting the Wnt/β-catenin signaling pathway in cancer. J Hematol Oncol. 2020;13(1):165. 33276800 10.1186/s13045-020-00990-3 PMC7716495

[25] Colozza G, Koo BK. Wnt/β-catenin signaling: structure, assembly and endocytosis of the signalosome. Dev Growth Differ. 2021;63(3):199‐218. 33619734 10.1111/dgd.12718 PMC8251975

[26] Komiya Y, Habas R. Wnt signal transduction pathways. Organogenesis. 2008;4(2):68‐75. 19279717 10.4161/org.4.2.5851 PMC2634250

[27] Bond CE, McKeone DM, Kalimutho M, et al RNF43 and ZNRF3 are commonly altered in serrated pathway colorectal tumorigenesis. Oncotarget. 2016;7(43):70589‐70600. 27661107 10.18632/oncotarget.12130 PMC5342576

[28] Giannakis M, Hodis E, Jasmine Mu X, et al RNF43 is frequently mutated in colorectal and endometrial cancers. Nat Genet. 2014;46(12):1264‐1266. 25344691 10.1038/ng.3127 PMC4283570

[29] Zhao H, Ming T, Tang S, et al Wnt signaling in colorectal cancer: pathogenic role and therapeutic target. Mol Cancer. 2022;21(1):144. 35836256 10.1186/s12943-022-01616-7 PMC9281132

[30] Werner J, Boonekamp KE, Zhan T, Boutros M. The roles of secreted Wnt ligands in cancer. Int J Mol Sci. 2023;24(6):5349. 36982422 10.3390/ijms24065349 PMC10049518

[31] Zhou Y, Huang Y, Cao X, et al WNT2 promotes cervical carcinoma metastasis and induction of epithelial-mesenchymal transition. PLoS One. 2016;11(8):e0160414. 27513465 10.1371/journal.pone.0160414 PMC4981407

[32] Xue W, Cai L, Li S, et al WNT ligands in non-small cell lung cancer: from pathogenesis to clinical practice. Discov Oncol. 2023;14(1):136. 37486552 10.1007/s12672-023-00739-7 PMC10366069

[33] Sastre-Perona A, Santisteban P. Role of the wnt pathway in thyroid cancer. Front Endocrinol (Lausanne). 2012;3:31. 22645520 10.3389/fendo.2012.00031 PMC3355838

[34] Ely KA, Bischoff LA, Weiss VL. Wnt signaling in thyroid homeostasis and carcinogenesis. Genes (Basel). 2018;9(4):204. 29642644 10.3390/genes9040204 PMC5924546

[35] Dong T, Zhang Z, Zhou W, et al WNT10A/beta–catenin pathway in tumorigenesis of papillary thyroid carcinoma. Oncol Rep. 2017;38(2):1287‐1294. 28677753 10.3892/or.2017.5777

[36] Sastre-Perona A, Riesco-Eizaguirre G, Zaballos MA. Santisteban P. beta-catenin signaling is required for RAS-driven thyroid cancer through PI3K activation. Oncotarget. 2016;7(31):49435‐49449. 27384483 10.18632/oncotarget.10356 PMC5226519

[37] Mackie EJ . Molecules in focus: tenascin-C. Int J Biochem Cell Biol. 1997;29(10):1133‐1137. 9438376 10.1016/s1357-2725(97)00031-9

[38] Flück M, Tunc-Civelek V, Chiquet M. Rapid and reciprocal regulation of tenascin-C and tenascin-Y expression by loading of skeletal muscle. J Cell Sci. 2000;113(Pt 20):3583‐3591. 11017874 10.1242/jcs.113.20.3583

[39] Chen S, Fu H, Wu S, et al Tenascin-C protects against acute kidney injury by recruiting Wnt ligands. Kidney Int. 2019;95(1):62‐74. 30409456 10.1016/j.kint.2018.08.029 PMC6320278

[40] Hendaoui I, Tucker RP, Zingg D, Bichet S, Schittny J, Chiquet-Ehrismann R. Tenascin-C is required for normal Wnt/β-catenin signaling in the whisker follicle stem cell niche. Matrix Biol. 2014;40:46‐53. 25196097 10.1016/j.matbio.2014.08.017

[41] Saupe F, Schwenzer A, Jia Y, et al Tenascin-C downregulates wnt inhibitor dickkopf-1, promoting tumorigenesis in a neuroendocrine tumor model. Cell Rep. 2013;5(2):482‐492. 24139798 10.1016/j.celrep.2013.09.014

[42] Giblin SP, Midwood KS. Tenascin-C: form versus function. Cell Adh Migr. 2015;9(1–2):48‐82. 25482829 10.4161/19336918.2014.987587 PMC4422809

[43] Dhaouadi S, Bouhaouala-Zahar B, Orend G. Tenascin-C targeting strategies in cancer. Matrix Biol. 2024;130:1‐19. 38642843 10.1016/j.matbio.2024.04.002

[44] Bartoschek M, Oskolkov N, Bocci M, et al Spatially and functionally distinct subclasses of breast cancer-associated fibroblasts revealed by single cell RNA sequencing. Nat Commun. 2018;9(1):5150. 30514914 10.1038/s41467-018-07582-3 PMC6279758

[45] Furuhashi S, Morita Y, Matsumoto A, et al Tenascin C in pancreatic cancer-associated fibroblasts enhances epithelial mesenchymal transition and is associated with resistance to immune checkpoint inhibitor. Am J Cancer Res. 2023;13(11):5641‐5655. 38058842 PMC10695794

[46] Oskarsson T, Acharyya S, Zhang XH, et al Breast cancer cells produce tenascin C as a metastatic niche component to colonize the lungs. Nat Med. 2011;17(7):867‐874. 21706029 10.1038/nm.2379 PMC4020577

[47] Puram SV, Tirosh I, Parikh AS, et al Single-Cell transcriptomic analysis of primary and metastatic tumor ecosystems in head and neck cancer. Cell. 2017;171(7):1611‐1624.e24. 29198524 10.1016/j.cell.2017.10.044 PMC5878932

[48] Lee SE, Park S, Yi S, et al Unraveling the role of the mitochondrial one-carbon pathway in undifferentiated thyroid cancer by multi-omics analyses. Nat Commun. 2024;15(1):1163. 38331894 10.1038/s41467-024-45366-0 PMC10853200

[49] Tang Z, Li C, Kang B, Gao G, Li C, Zhang Z. GEPIA: a web server for cancer and normal gene expression profiling and interactive analyses. Nucleic Acids Res. 2017;45(W1):W98‐W102. 28407145 10.1093/nar/gkx247 PMC5570223

[50] Cerami E, Gao J, Dogrusoz U, et al The cBio cancer genomics portal: an open platform for exploring multidimensional cancer genomics data. Cancer Discov. 2012;2(5):401‐404. 22588877 10.1158/2159-8290.CD-12-0095 PMC3956037

[51] Gao J, Aksoy BA, Dogrusoz U, et al Integrative analysis of complex cancer genomics and clinical profiles using the cBioPortal. Sci Signal. 2013;6(269):pl1. 23550210 10.1126/scisignal.2004088 PMC4160307

[52] de Bruijn I, Kundra R, Mastrogiacomo B, et al Analysis and visualization of longitudinal genomic and clinical data from the AACR project GENIE biopharma collaborative in cBioPortal. Cancer Res. 2023;83(23):3861‐3867. 37668528 10.1158/0008-5472.CAN-23-0816 PMC10690089

[53] ggplot2 : Elegant Graphics for Data Analysis . Version 2nd 2016. Springer. Springer International Publishing: Imprint: Springer; 2016. 10.1007/978-3-319-24277-4

[54] Hanzelmann S, Castelo R, Guinney J. GSVA: gene set variation analysis for microarray and RNA-seq data. BMC Bioinformatics. 2013;14:7. 23323831 10.1186/1471-2105-14-7 PMC3618321

[55] Liberzon A, Birger C, Thorvaldsdottir H, Ghandi M, Mesirov JP, Tamayo P. The Molecular Signatures Database (MSigDB) hallmark gene set collection. Cell Syst. 2015;1(6):417‐425. 26771021 10.1016/j.cels.2015.12.004 PMC4707969

[56] Li T, Fu J, Zeng Z, et al TIMER2.0 for analysis of tumor-infiltrating immune cells. Nucleic Acids Res. 2020;48(W1):W509‐W514. 32442275 10.1093/nar/gkaa407 PMC7319575

[57] Racle J, Gfeller D. EPIC: a tool to estimate the proportions of different cell types from bulk gene expression data. Methods Mol Biol. 2020;2120:233‐248. 32124324 10.1007/978-1-0716-0327-7_17

[58] Viloria K, Hill NJ. Embracing the complexity of matricellular proteins: the functional and clinical significance of splice variation. Biomol Concepts. 2016;7(2):117‐132. 27135623 10.1515/bmc-2016-0004

[59] Hartmann H . Supplementary Material for “Tenascin-C potentiates Wnt signaling in thyroid cancer”. doi:10.5281/zenodo.14713514.

[60] Lowy CM, Oskarsson T. Tenascin C in metastasis: a view from the invasive front. Cell Adh Migr. 2015;9(1–2):112‐124. 25738825 10.1080/19336918.2015.1008331 PMC4422797

[61] Elyada E, Bolisetty M, Laise P, et al Cross-Species single-cell analysis of pancreatic ductal adenocarcinoma reveals antigen-presenting cancer-associated fibroblasts. Cancer Discov. 2019;9(8):1102‐1123. 31197017 10.1158/2159-8290.CD-19-0094 PMC6727976

[62] Unterleuthner D, Neuhold P, Schwarz K, et al Cancer-associated fibroblast-derived WNT2 increases tumor angiogenesis in colon cancer. Angiogenesis. 2020;23(2):159‐177. 31667643 10.1007/s10456-019-09688-8 PMC7160098

[63] Aizawa T, Karasawa H, Funayama R, et al Cancer-associated fibroblasts secrete Wnt2 to promote cancer progression in colorectal cancer. Cancer Med. 2019;8(14):6370‐6382. 31468733 10.1002/cam4.2523 PMC6797671

[64] Hancox RA, Allen MD, Holliday DL, et al Tumour-associated tenascin-C isoforms promote breast cancer cell invasion and growth by matrix metalloproteinase-dependent and independent mechanisms. Breast Cancer Res. 2009;11(2):R24. 19405959 10.1186/bcr2251 PMC2688953

[65] Geleta B, Tout FS, Lim SC, et al Targeting Wnt/tenascin C-mediated cross talk between pancreatic cancer cells and stellate cells via activation of the metastasis suppressor NDRG1. J Biol Chem. 2022;298(3):101608. 35065073 10.1016/j.jbc.2022.101608 PMC8881656

[66] Wild SL, Elghajiji A, Grimaldos Rodriguez C, Weston SD, Burke ZD, Tosh D. The canonical Wnt pathway as a key regulator in liver development, differentiation and homeostatic renewal. Genes (Basel). 2020;11(10):1163. 33008122 10.3390/genes11101163 PMC7599793

[67] Kuriyama N, Duarte S, Hamada T, Busuttil RW, Coito AJ. Tenascin-C: a novel mediator of hepatic ischemia and reperfusion injury. Hepatology. 2011;54(6):2125‐2136. 21898491 10.1002/hep.24639 PMC3230719

[68] Kato H, Duarte S, Miller MG, Busuttil RW, Coito AJ. Overproduction of tenascin-C driven by lipid accumulation in the liver aggravates hepatic ischemia/reperfusion injury in steatotic mice. Liver Transpl. 2019;25(2):288‐301. 30358115 10.1002/lt.25365 PMC6355355

