# Correction to: “Tenascin-C Potentiates Wnt Signaling in Thyroid Cancer”

**DOI:** 10.1210/endocr/bqaf053

**Published:** 2025-03-20

In the above-named article by Hartmann HA, Loberg MA, Xu GJ, Schwarzkopf AC, Chen S-C, Phifer CJ, Caroland K, Chen H-C, Diaz D, Tigue ML, Hesterberg AB, Gallant J-N, Shaddy SM, Sheng Q, Netterville JL, Rohde SL, Solórzano CC, Bischoff LA, Baregamian N, Hurley PJ, Murphy BA, Choe JH, Huang EC, Ye F, Lee E, and Weiss VL (*Endocrinology*. 2025; 166(3); doi: 10.1210/endocr/bqaf030), there was an error in Figure 6.

In the originally published article, in Figure 6, the image in panel A was unintentionally duplicated in panel B. The authors identified this inadvertent error and provided a corrected replacement for Figure 6, which is included in the corrected article and is shown below. The authors state that the error does not imply any changes to the scientific conclusions in the article, and the caption remains correct with the replacement image.

The article has been corrected online.

Original Figure 6:

**Figure:**
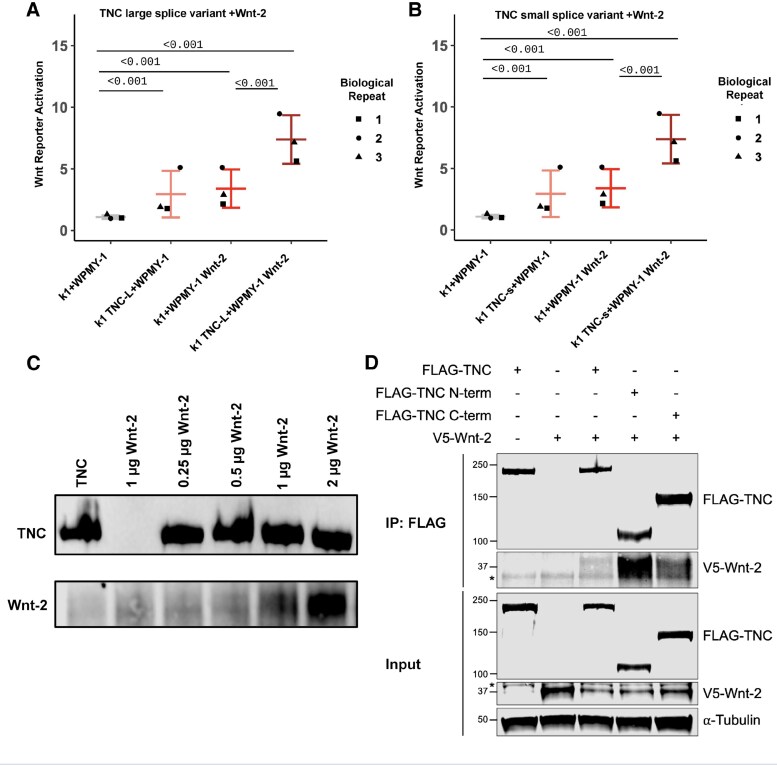


Corrected Figure 6:

**Figure:**
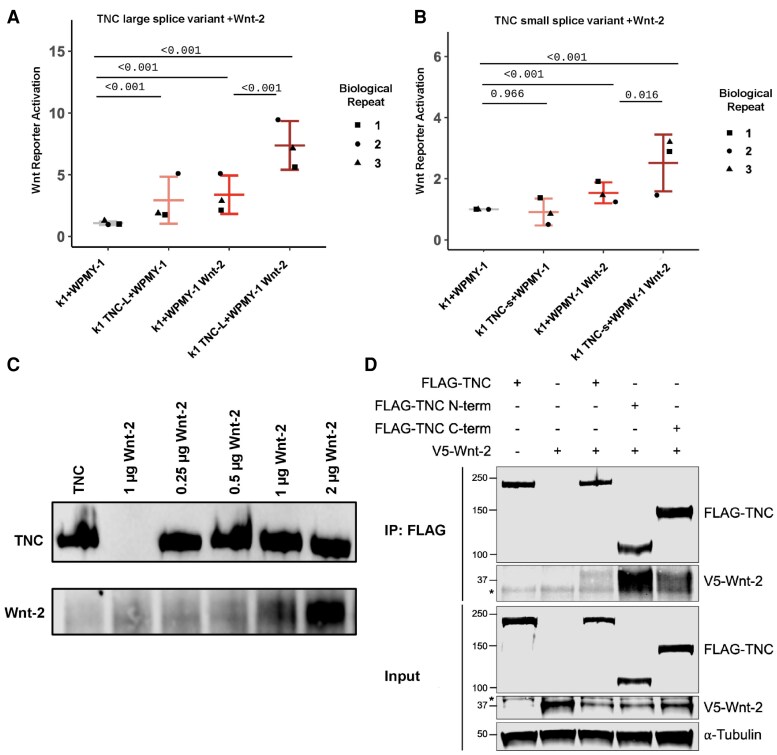


doi: 10.1210/endocr/bqaf030

